# Cranial dermoid cyst with long-term development treated by ethanol sclerotherapy: a case report

**DOI:** 10.1080/23320885.2020.1835485

**Published:** 2020-10-26

**Authors:** Takeshi Kitazawa, Masato Shiba, Hiroyuki Nagaya, Shunsuke Yuzuriha

**Affiliations:** a Department of Plastic and Reconstructive Surgery, Matsunami General Hospital, Gifu, Japan; b Department of Plastic and Reconstructive Surgery, Shinshu University School of Medicine and Graduate School of Medicine, Matsumoto, Japan

**Keywords:** Dermoid cyst, sclerotherapy, long term, orbit

## Abstract

Here, we describe the case of an 80-year-old woman who presented with cranial dermoid cyst causing orbital disfigurement. The cyst was treated successfully with ethanol sclerotherapy and has shown no growth for 1 year.

## Introduction

Dermoid cysts are benign subcutaneous tumors, which may be present at birth and develop gradually over time. Most periorbital dermoid cysts are easily detected and the patient presents for surgery for cosmetic reasons in childhood or adolescence. We describe herein an extremely rare case of dermoid cyst in an 80-year-old woman, causing orbital compression and treated by sclerotherapy.

## Case presentation

**Figure 1.**
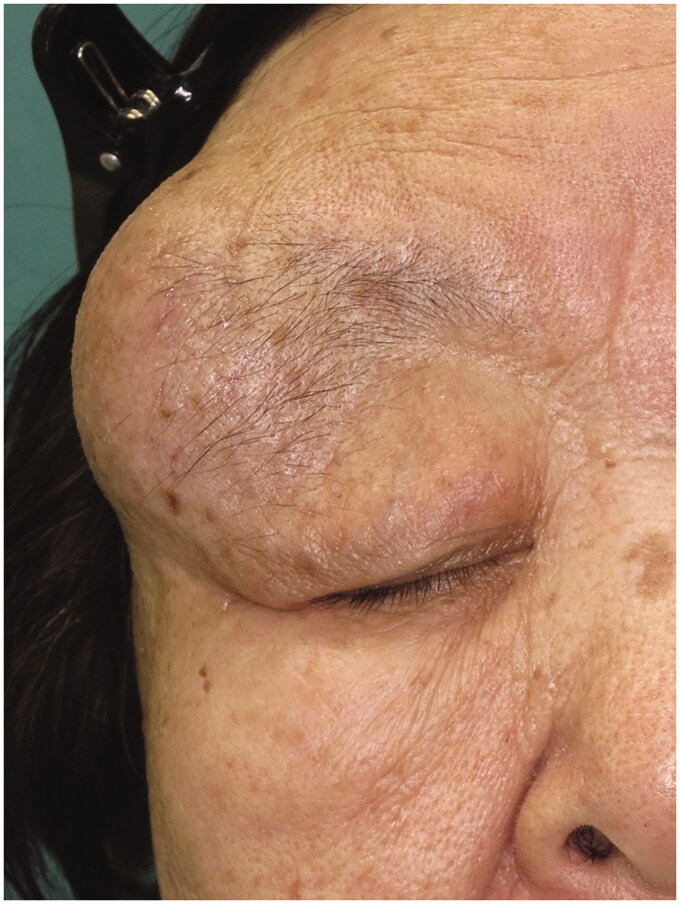
Clinical view of the lesion at first presentation.

An 80-year-old woman was referred to our department with a 50-year history of a slow-growing, painless cyst in the right lateral orbital region. The cyst had been aspirated at regular intervals by previous physicians, but always re-expanded shortly thereafter. Examination revealed a single, well-defined, elastic hard mass fixed to the orbit, with normal-appearing surrounding skin (Figure 1). Mechanical ptosis due to the cyst, 6 mm of proptosis and slight hypotropia were observed in the right eye, but no abnormal findings were detected in the lens and fundus. The patient had no complaints of diplopia.

**Figure 2.**
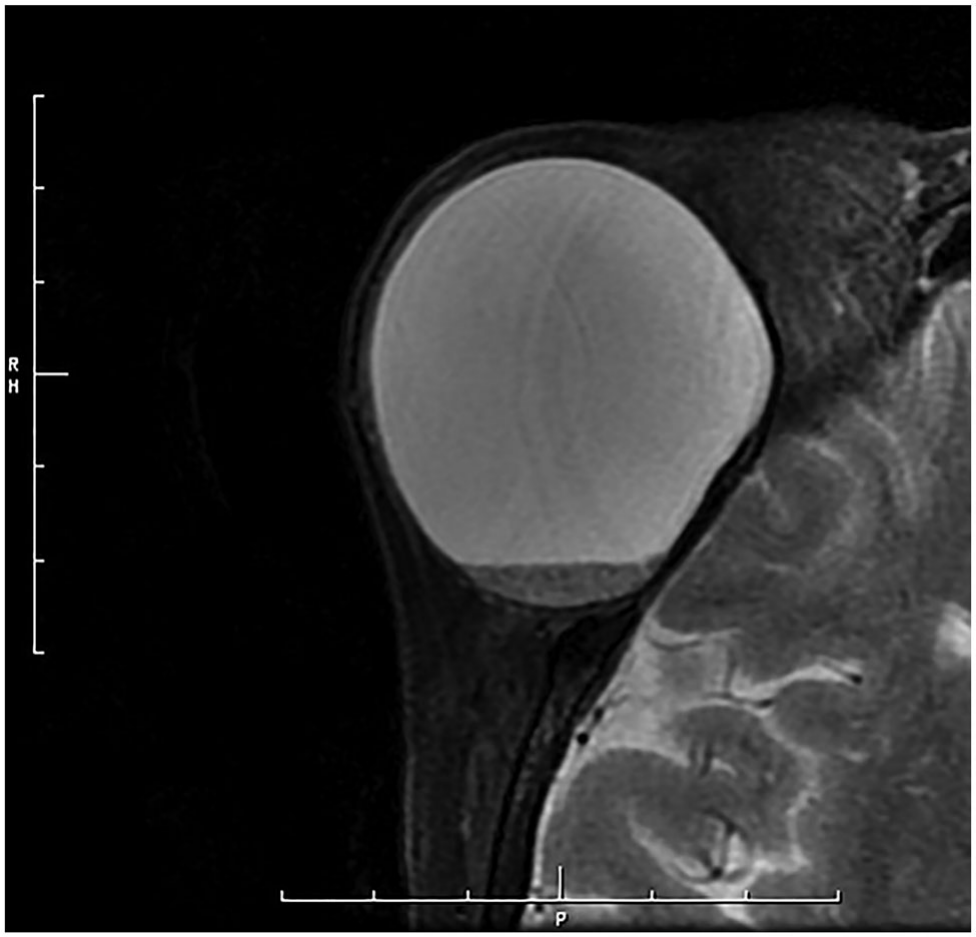
T2-weighted MRI demonstrates a round, high-intensity mass.

**Figure 3.**
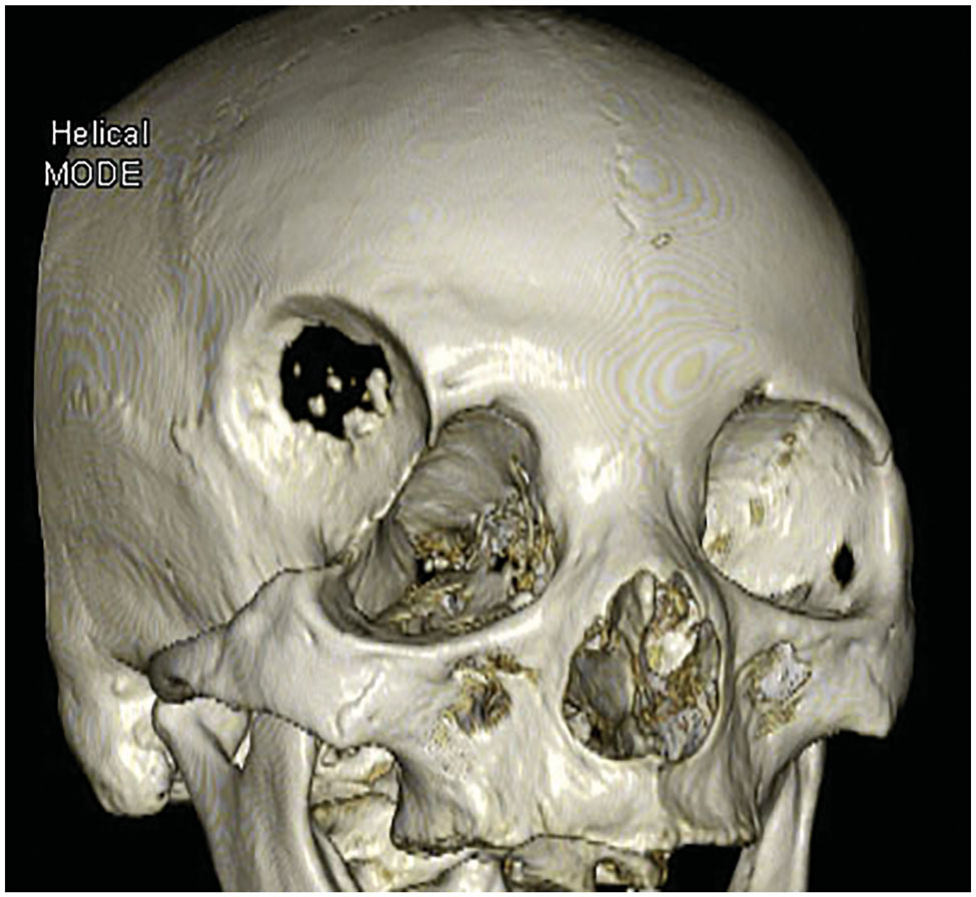
Computed tomography shows full-thickness bone erosion at the right frontosphenoidal suture and orbital disfigurement.

Magnetic resonance imaging (MRI) demonstrated a round mass measuring 45 × 43 × 45 mm superolateral to the right orbit, showing low intensity on T1-weighted imaging and high intensity on T2-weighted imaging with no enhancement by gadolinium (Figure 2). Computed tomography (CT) showed deformation of the right orbit and bone erosion centering on the frontosphenoidal suture (Figure 3).

On the basis of these findings, we diagnosed the lesion as dermoid cyst, and proposed surgical removal of the cyst and boney reconstruction with hydroxyapatite paste. However, the patient firmly declined such an operation and wished to continue periodic aspiration.

**Figure 4.**
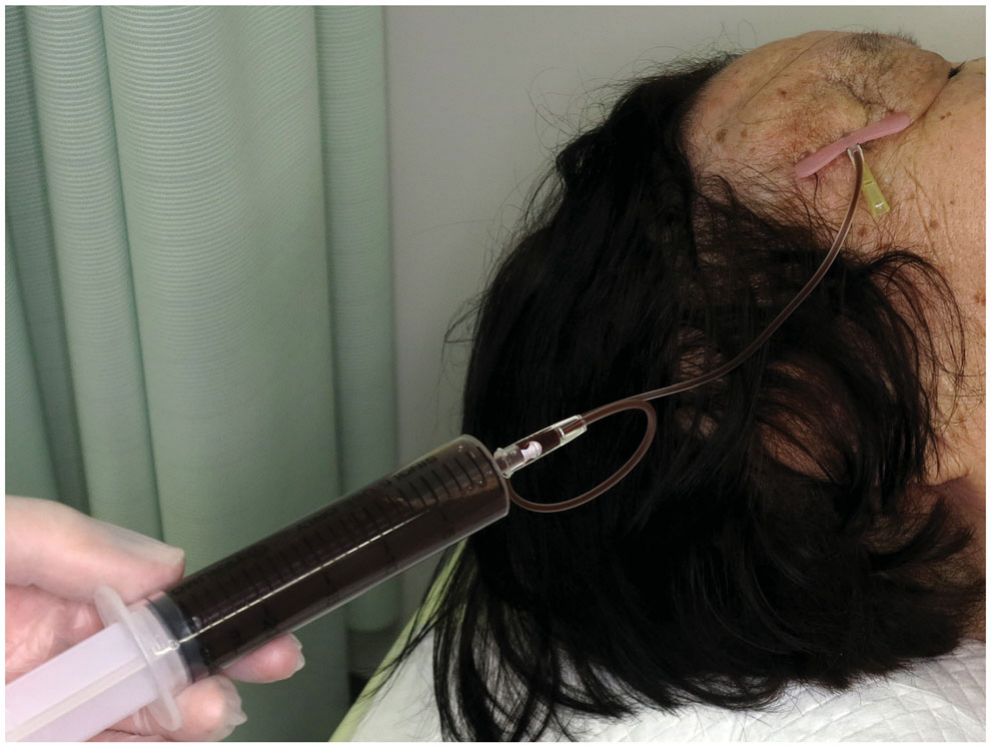
Dark but uncontaminated fluid is aspirated from the cyst before sclerotherapy.

Aspiration was performed 3 times within the 3 years after her first visit. The aspirated fluid was dark-brown and serous in nature, without debris or contamination, and fluid volumes were 34, 30, and 37 mL, respectively (Figure 4). The fluid contained a few histiocytes, but no atypical cells were detected on cytological analysis. As MRI revealed a monocystic, homogeneous, well-circumscribed lesion, sclerotherapy was considered potentially effective against the cyst, and was performed at the time of the fourth aspiration after obtaining informed consent from the patient.

The cyst was punctured with an 18-G needle under local anesthesia and fluid was completely aspirated. After the cyst cavity was washed with 10 mL of saline, 10 mL of 1% lidocaine containing 1:100,000 epinephrine was instilled and left in place for 2–3 min. Next, the lidocaine was aspirated completely, and 10 mL of absolute ethanol was instilled and left in place for 1 min. The ethanol was then completely aspirated, and the cavity was washed again with 10 mL of saline.

Three months after the first sclerotherapy, the cyst had decreased in size and 15 mL of clear, yellow fluid was aspirated. Second and third sclerotherapies were performed in the same manner, at intervals of 3 months.

**Figure 5.**
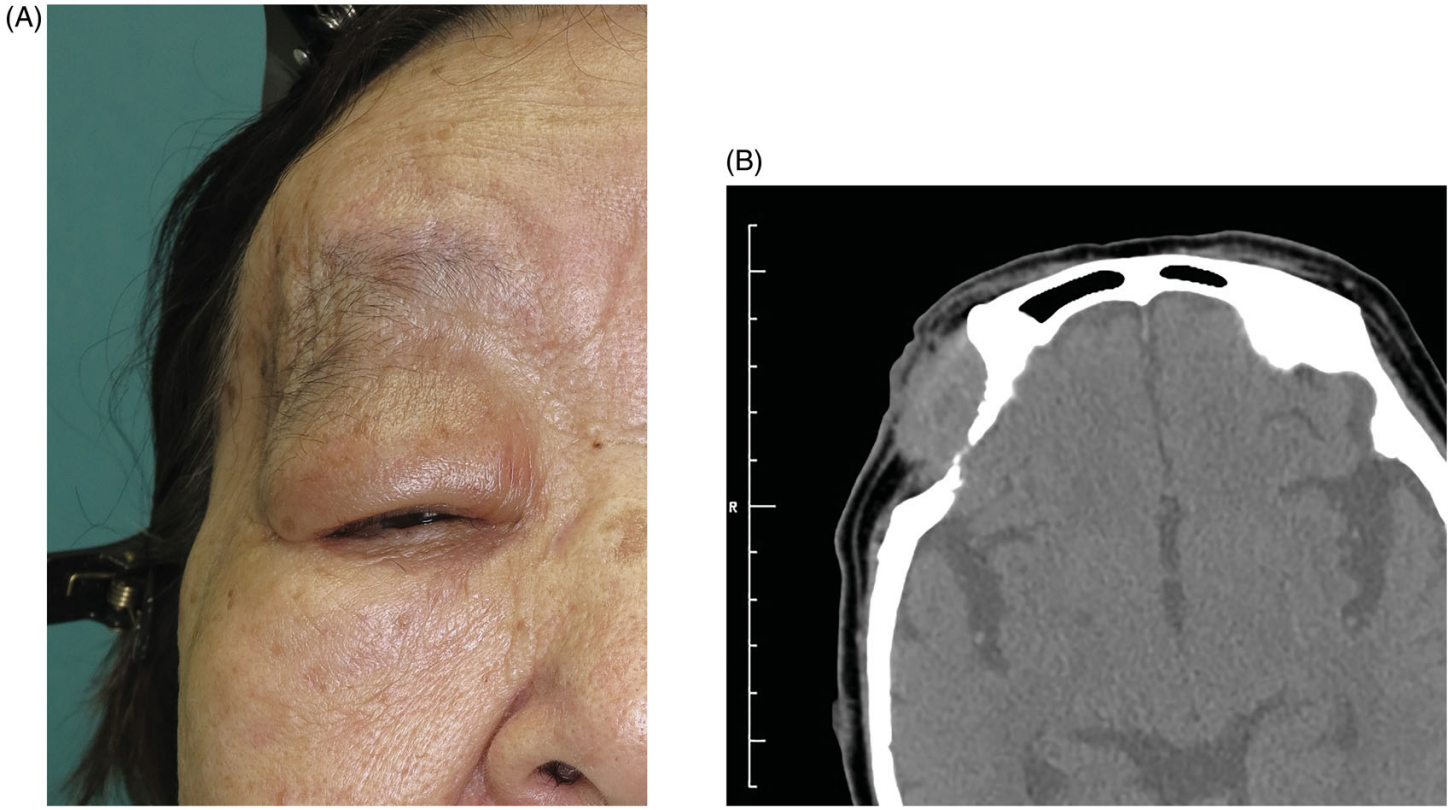
Gross appearance (A) and computed tomography (B) at 1 year after final sclerotherapy. The cyst did not disappear, but shrank to become inconspicuous in appearance.

**Figure 6.**
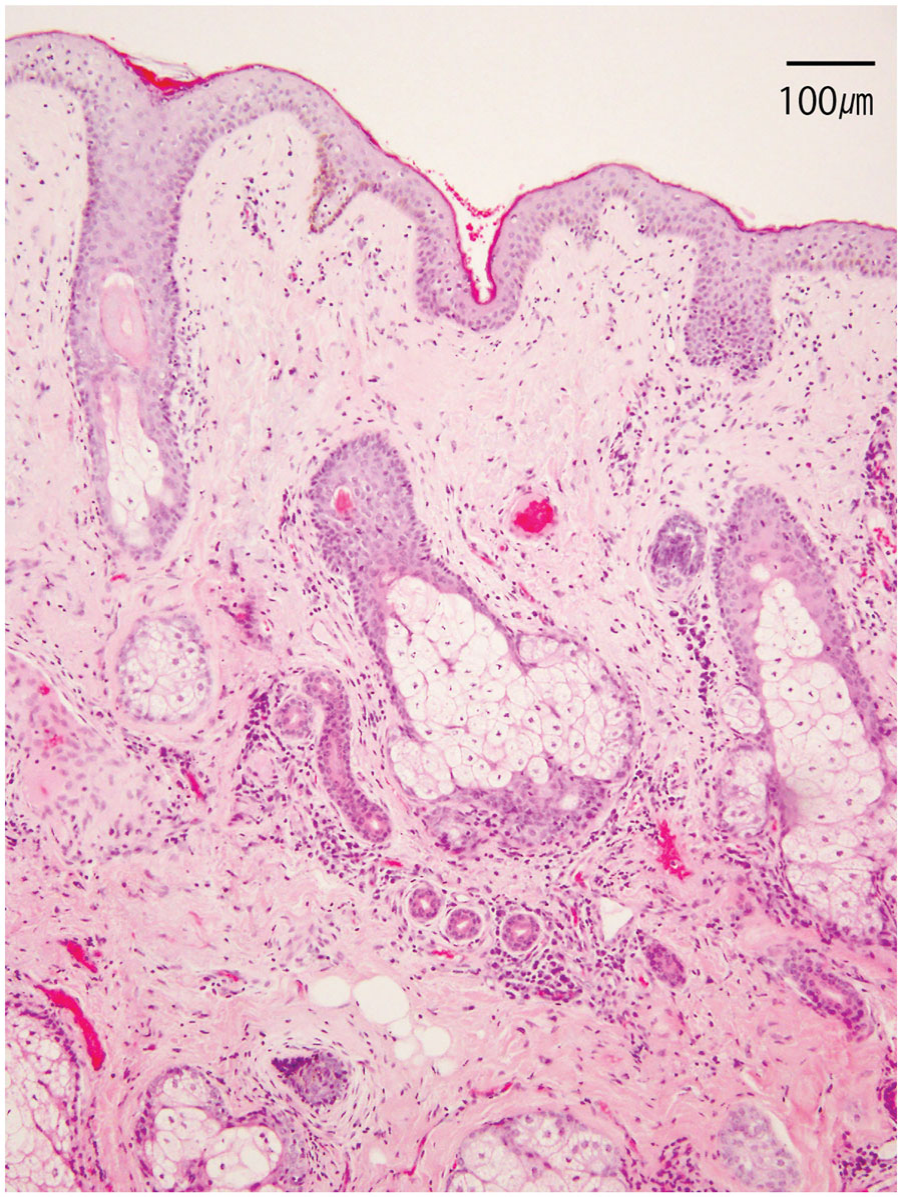
Photomicrograph of a portion of cyst wall demonstrating squamous epithelium lining concomitant with sebaceous and sweat glands (hematoxylin and eosin stain).

Six months after the last sclerotherapy, the cyst had shrunk in size to 26 × 15 × 35 mm (Figure 5). However, the patient complained of visual field disturbance due to the redundant skin. Blepharoplasty concomitant with incisional biopsy of the cyst was performed. Histopathological examination showed that the specimen comprised dermis with well-developed sweat and sebaceous glands, compatible with dermoid cyst (Figure 6). No re-expansion was seen during a 1-year follow-up after the last sclerotherapy.

## Discussion

Dermoid cysts are benign, soft-tissue tumors that develop from entrapment of surface ectoderm along the lines of embryonic fusion and have a capacity to grow. Eighty-four percent of dermoid cysts reportedly occur in the head or neck [1], with the periorbital region as the most common [2]. As dermoid cysts are considered congenital, approximately 90% of cysts around the cranium are detected and treated during the first decade of life [3,4]. Although dermoid cysts are histologically benign and some remain unchanged in size, the possibility of spontaneous rupture or infection remains, and gradual but continuous growth may cause pressure-related bone erosion. As a result, surgical extirpation is considered the optimum treatment [5].

In the present case, the origin was assumed to be the frontosphenoidal suture because of the full-thickness bony erosion at that site. Located deep to the temporalis muscle, the patient might have been unaware of the cyst until her 30 s. Through the 80 years of enlargement, not only full-thickness bone erosion, but orbital disfigurement was noted.

To the best of our knowledge, only two reports have described sclerotherapy for dermoid cyst [6,7]. Golden et al. [6] used sodium tetradecyl sulfate (STS) and absolute ethanol as sclerosants for 2 cases. Naik et al. [7] used foamed STS in 4 cases.

Ethanol sclerotherapy is widely performed for cystic masses. Baker’s cyst, branchial cleft cyst, thyroglossal duct cyst [8], and seroma [9] have reportedly been treated successfully using this method. Ethanol causes inflammatory reactions involving endothelial cells of the cyst wall, leading to fibrosis and cyst closure. Sclerotherapy using absolute ethanol has the benefit of technical simplicity and low cost compared to other sclerosants [10].

Even though all 7 cases (including the present case) were treated successfully, various limitations of sclerotherapy for dermoid cyst should be recognized. One is that the material within the dermoid cyst must be aspiratible through the needle or catheter. The contents of dermoid cysts, comprising substances such as keratin, sebum, and fat, is often pultaceous and difficult to aspirate. Although the exact ratio of dermoid cysts for which sclerotherapy can be applied has remained unknown, Pushker et al. [11] stated that fluid-fluid levels indicative of liquid collection are seen within the cyst on CT in 24% cases of orbital dermoid cyst. Thus, approximately only one-quarter of dermoid cysts would react to sclerotherapy successfully.

As shown in our histological examinations, despite cyst reduction, the dermal component of the cyst wall still existed after sclerotherapy. This implies a risk of cyst re-expansion, representing another limitation of sclerotherapy. Furthermore, most dermoid cysts are detected early enough to be extirpated safely with low invasiveness during the first decade of life. These limitations and benefit of surgical extirpation seem to be why only very small numbers of case reports have described sclerotherapy for dermoid cysts.

Sclerotherapy may not eradicate dermoid cysts, but has potential to decrease cyst size. This treatment can thus be adopted for patients who cannot undergo operation for whatever reason, or as preoperative therapy to reduce the tumor volume.

While long-term follow-up is warranted, this case suggests the feasibility of sclerotherapy for dermoid cyst, but underlines the importance of early diagnosis and cyst removal without delay.

## Conclusion

Long-term development of cranial dermoid cyst can cause bone erosion and facial disfigurement. Although sclerotherapy was effective in this case, surgical extirpation during the early stage is currently preferred to prevent sequelae.
